# The effects of indoor plants and traffic noise on English reading comprehension of Chinese university students in home offices

**DOI:** 10.3389/fpsyg.2022.1003268

**Published:** 2022-09-29

**Authors:** Yuanyuan Zhang, Dayi Ou, Qiu Chen, Shengxian Kang, Guanhua Qu

**Affiliations:** ^1^School of Architecture, Huaqiao University, Xiamen, China; ^2^Xiamen Key Laboratory of Ecological Building Construction, Xiamen, China; ^3^State Key Lab of Subtropical Building Science, South China University of Technology, Guangzhou, China; ^4^Wenzhou Lucheng District Land Reserve and Transfer Center, Wenzhou, China; ^5^The Department of Building Environment and Energy Engineering, The Hong Kong Polytechnic University, Hong Kong, Hong Kong SAR, China; ^6^School of Architecture, Tianjin University, Tianjin, China

**Keywords:** COVID-19, home offices, audio-visual interaction, indoor plants, traffic noise, English reading comprehension

## Abstract

Owing to the COVID-19 pandemic, working from home promotes the importance of indoor environment qualities. With the settings and functions of home offices, an experiment was carried out to determine the interaction effects between indoor plants and traffic noise levels (TNLs) on the performance and environmental evaluations of English reading comprehension tasks (ERCTs) and the performance of short-term breaks. A sample of 22 Chinese university students (12 males and 10 females) took part in the experiment. Two visual conditions (with and without plants) and five TNLs (i.e., 35, 45, 50, 55, and 60 dBA TNL) were included. Participants’ accuracy rates, eye movements, mental workload, and feelings about the environment were collected. The mental fatigue recovery (MFR), visual fatigue recovery (VFR), anxiety recovery (AR), and unfriendly recovery (UR) were measured for the analysis of a 5-min short-term break. The results demonstrate (1) plants have significant effects on ERCTs and short-term breaks, especially at 45 and 50 dBA TNL; (2) the effects of TNLs on ERCTs’ eye movements and work environment satisfaction differ by the presence of plants, e.g., the average pupil diameter (APD), lighting and layout satisfaction; (3) The effects of indoor plants on ERCT differ by the range of TNLs. In conclusion, indoor plants are beneficial to home workers engaged in ERCT when TNL does not exceed 50 dBA. The current data highlight the importance of audio-visual interaction in home offices and provide insights into the interaction mechanism between indoor plants and traffic noise.

## Introduction

Indoor environment qualities in home offices are critical for modern people especially after the outbreak of the COVID-19 pandemic when working at home has been widely chosen as a coping strategy to reduce the risk of infection and protect health. Among them, the acoustic environment is crucial for home workers’ productivities and environmental perceptions (Yu and Kang, 2010; Torresin et al., 2021). The traffic noise has been widely considered a critical acoustic problem (Ouis, 2001; öhrström et al., 2006; Brink, 2011; Environmental Noise Guidelines for the World Health Organization, 2018; Torresin et al., 2022), which exerts serious threats to residents’ cognitive performance (Wei, 2020; Chen and Ou, 2021), subjective perceptions (Brink, 2011; Hegewald et al., 2020), and health (Environmental Noise Guidelines for the World Health Organization, 2018; Hegewald et al., 2020; Stansfeld et al., 2021). For example, traffic noise level (TNL) is associated with an increased risk of vascular dementia up to approximately 60 dB, where it levels off (Cantuaria et al., 2021). From 2001 to 2015, Shin et al. (2020) have investigated and found that for every 10 decibels increase, the risk of diabetes mellitus increases by 8%, and hypertension increases by 2% in Toronto. Exposure to 56–60 dBA road traffic noise is associated with psychological ill-health, measured by the General Health Questionnaire (Stansfeld et al., 2021). A 45 dBA has often been considered the critical recommended value at night (Environmental Noise Guidelines for the World Health Organization, 2018; Li and Siu-Kit, 2020). Guideline development group (GDG) also strongly recommends reducing TNLs below 53 dBA to avoid health effects and below 45 dBA to avoid sleep disturbance (Environmental Noise Guidelines for the World Health Organization, 2018). It is also recommended by Normenausschuss Akustik, Lärmminderung, und Schwingungstechnik (NALS) (VDI 2058–3, 2014) that noise level should be maintained below 55 dBA for predominantly mental activities and below 70 dBA for simple office activities. Wei has examined the influence of road traffic noise (30, 50, and 70 dBA) on English reading comprehension (easy, medium, and hard) of Chinese college students majoring in English and found that the scores of English reading comprehension with easy questions decreased with the TNL increased from 30 to 70 dBA (Wei, 2020). Overall, these studies highlight the necessity to reduce the TNL for participants’ health, subjective perceptions, and cognitive performance. Given that, the recommended TNL usually varies from 45 to 60 dBA, with 5 dBA a widely accepted step size (Environmental Noise Guidelines for the World Health Organization, 2018; Cantuaria et al., 2021). Consistent with relevant research, 45, 50, 55, and 60 dBA TNLs were also considered in this study. A quiet environment without traffic noise was regarded as a control.

Audio-visual interactions have been widely employed to improve the acoustic quality of the environment, such as noise barriers and green areas (Li and Siu-Kit, 2020). Vegetation-covered barriers increase preconceptions of noise attenuation and aesthetic preferences (Chen and Ping, 2005). Viewing plants may reduce traffic noise annoyance (Angel and Donka, 2015). Hong et al., have found that barriers’ preconceived noise attenuations are more critical than esthetic preferences for the overall choice at 55 dBA and reversed at 65 dBA (Joo and Jin, 2014). In non-focused conditions, increasing pleasantness by improving the visual design of barriers (such as vegetations) would be more effective than increasing noise barrier height (Gemma et al., 2017). Researchers have found that nature features may be indicators of a tranquil environment (Pheasant et al., 2008; Gemma et al., 2017). A higher sound pressure level may be associated with a higher psychological demand for natural features (Pheasant et al., 2008; Annu et al., 2020). The requirement for acoustic environments would also be higher for complex tasks (e.g., English reading comprehension) than simple tasks (Haapakangas et al., 2020; Lou and Ou, 2020). However, comparative studies remain narrow in focus dealing only with accuracy rates and subjective perceptions. A more systematic and theoretical analysis is required to uncover the interaction mechanism between indoor plants and traffic noise on complex tasks.

Pupil diameter has been generally considered a valid cognitive load index (Beatty, 1982; Daniel and Jackson, 2018; Krejtz et al., 2018). A higher average pupil diameter (APD) value is associated with a higher workload level. Pupil diameter is sensitive to the demanded cognitive processing and factors unrelated to task difficulty (Debue and van de Leemput, 2014; Krejtz et al., 2018), such as the attention of auditory background (Liao et al., 2016; Alexandre et al., 2018). Traffic noise is associated with pupil dilation *via* cardiac parasympathetic withdrawal (Jamie et al., 2009). Visual information processing is disabled at saccades and abled at fixations. Longer saccades were often accompanied by shorter APD (Velichkovsky et al., 2005). Perceptions during the break are also observed to explore whether indoor plants performed similarly after different conditions. In this study, the APD and total amplitude of saccades (TAS) were observed to explore the interaction between indoor plants and traffic noise on the ERCT.

Biophilia is a biological phenomenon of escaping the urban environment to nature by improving mood and mental and physical responses (Fromm, 1964). Plants have been widely used in indoor environments for psychological and physiological benefits (Mcsweeney et al., 2015; Chorong et al., 2016; Lee, 2020; Aydogan and Cerone, 2021). Epipremnum aureum is the most common plant in the indoor environment (Lee, 2020), removing volatile organic carcinogens at a reasonable rate (Zhang et al., 2019). Interaction with plants has been observed to link with suppressing sympathetic nervous system activity and diastolic blood pressure, promoting comfortable, soothed, and natural feelings (Lee et al., 2015). During the COVID-19 pandemic, occupants have had less natural exposure to outdoor environments. Dzhambov et al. (2020) have performed an online survey in times of physical isolation at home and reported that students have experienced less depression and anxiety when exposed to more greenery. A correlation between viewing indoor plants and decreased nervousness and anxiety have also been observed by Chang and Chen, who simulate six environments in an office by Photo Impact 5.0 to study human response to window views and indoor plants (Chen and Ping, 2005). It is evident that indoor plants help promote productivity (Ke-Tsung and Li-Wen, 2019) and concentration (Virginia et al., 1996; Ruth et al., 2011) and suppress stress (Virginia et al., 1996). McSweeney et al. (2021) have found that indoor plants are positively associated with post-task assessments. The presence of plants increases positive emotions and reduces physical discomfort (Ke-Tsung and Li-Wen, 2019). Breaks with plants accelerates stress restoration (Tina et al., 2009; Ke-Tsung, 2018) and elevates perceptions (with decreased anxiety and fatigue and increased satisfaction) (Park and Mattson, 2009). In contrast, Kalevi et al. (2017) have found through a survey that indoor plants are negatively associated with the number of breaks and positively associated with workload. Thus, current research fails to answer clearly whether plants are indeed beneficial for work performance.

In a word, traffic noise may seriously threaten participants’ work performance and environmental perceptions (Simone et al., 2020). Indoor plants are commonly set in a home for preference, air quality improvement, and ornament. However, based on existing research, it is still uncertain whether plants are actually beneficial for work and short-term breaks. This study has focused on the English reading comprehension task (ERCT) because of its importance and specificity for Chinese university students (Lou and Ou, 2020). English reading comprehension is a necessary skill for almost every university student. Moreover, English as the dominant language in scientific literature, ERCTs are always considered the actual daily work, especially for university students from non-English countries. However, much of the recent literature has not analyzed the impacts of audio-visual interaction on this specific task (ERCT) in home offices.

The purpose of the present study is twofold. The first aim was to explore the effects of indoor plants and TNLs on participants’ ERCT performance and environmental perceptions in a home office. Work performance, mental workload, feelings about the acoustic environment, and feelings about the non-acoustic environment were examined. The second aim was to further analyze the effects of indoor plants and TNLs on a 5-min short-term break by considering MFR, VFR, anxiety recovery, and unfriendly recovery.

## Materials and methods

### Laboratory room

**Figure 1:**
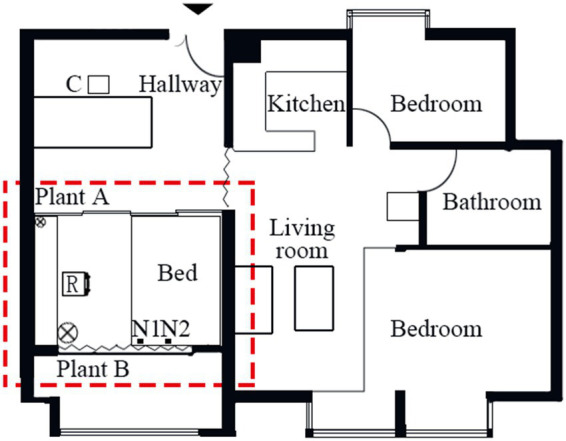
The layout of the residential unit. The area marked by a dashed line is the home office. R, C, and N1-N2 represent the locations of the test workstation, control console, and traffic noise generators, respectively. Indicates the positions of plants.

The experiment was carried out in a home office with a size of 3.9 m (length) × 3.0 m (width) on the 19th floor of a 29-story residential building in Xiamen city, China. As shown in Figure 1, one workstation (R) was used as a test position in the middle of the room. The control console (C) was in a neighbor’s room. Two loudspeakers (Genelec 8010A) (N1 and N2) were set near the window to play traffic noise. Illuminance was set to about 490 lux on the table. On-site measurements of the light environment showed that the unified glare rating (UGR) was 12.4 (<19), and the desktop illuminance was 543 lux (>500 lux) [referring to ISO 8995-1:2002 (ISO 8995-1, 2002)]. The indoor temperature during the experiment varied between 24 and 26°C, and the relative humidity ranged from 50 to 70%. The background noise level (BNL) was at around 35 dBA by AWA 6291 sound level meter. The sound level meter was placed at the test position, and had a height of 1.2 m above the indoor ground. The measurement lasted 12 min. In the process of measurement, air-conditioning, television, and other large electronic equipment were powered off.

### Experimental conditions

Two visual conditions (the home office with and without plants) and five acoustic conditions were chosen. A total of 10 experimental conditions are presented in Table 1. The five acoustic conditions included a quiet condition and four traffic noise conditions. The quiet condition (BNL = 35 dBA) was the condition with the room background noise level of about 35 dBA, which was without traffic noise. Previous studies have widely used the four traffic noise levels (TNL = 45, 50, 55, and 60 dBA). The reasons for the chosen TNLs were seen in the first paragraph of Section 1 Introduction. The quiet environment without traffic noise (BNL = 35 dBA) was used as the control condition. Two loudspeakers (Genelec 8010A) (N1 and N2) were powered on to generate traffic noise. The traffic noise materials were prepared before the experiment. Four traffic noise conditions were achieved by adjusting the volume of the two loudspeakers. AWA 6291 sound level meter was set at 1.2 m above the indoor ground at the test position. The equivalent continuous A-weighted sound pressure levels of the road TNLs during each acoustic condition were set to be very close to their target values, respectively (i.e., 45, 50, 55, and 60 dBA).

**Table 1:** Information of 10 experimental conditions.

Condition	Descriptions	Condition	Descriptions
P_B_35	With plants at BNL = 35 dBA	NP_B_35	Without plants at BNL = 35 dBA
P_T_45	With plants at TNL = 45 dBA	NP_T_45	Without plants at TNL = 45 dBA
P_T_50	With plants at TNL = 50 dBA	NP_T_50	Without plants at TNL = 50 dBA
P_T_55	With plants at TNL = 55 dBA	NP_T_55	Without plants at TNL = 55 dBA
P_T_60	With plants at TNL = 60 dBA	NP_T_60	Without plants at TNL = 60 dBA

The background noise level of the home office was about 35 dBA. “P” and “NP” represent the conditions with plants and without plants, respectively. “B” represents a quiet condition without traffic noise, and “T” represents conditions with traffic noise.

**Figure 2:**
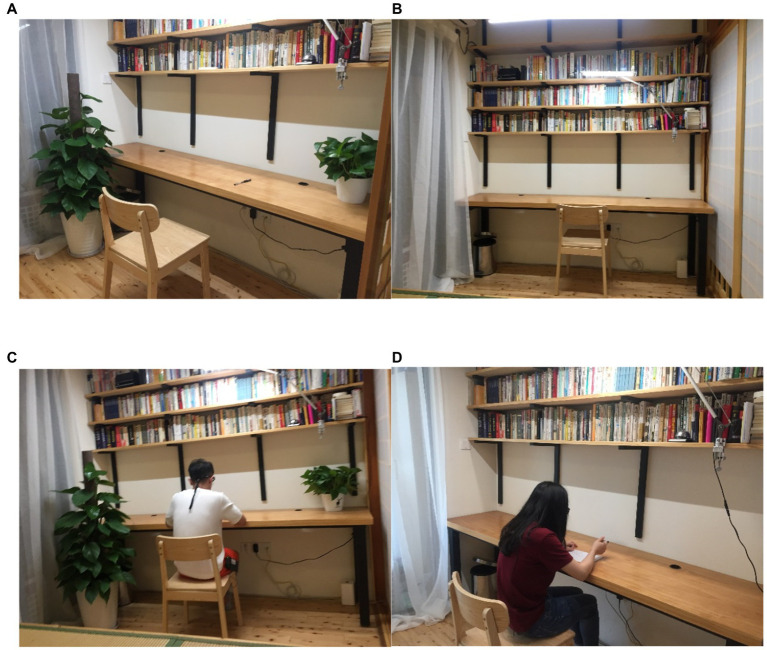
**(A,C)** The home office with plants. **(B,D)** The home office without plants.

Representative photos of the home office are shown in Figure 2. As seen in Figures 2A,C, two potted plants (Epipremnum aureum) of different sizes were used as a treatment to improve visual conditions. The smaller plant with a height of 30 cm and a width of 40 cm was located on the table of the workstation, and the larger one with a height of 150 cm and a width of 50 cm was set on the floor (see Figures 1, 2B,D) show conditions without plants.

The traffic noise materials were prepared before the experiment, recorded at 1.5 m from the road’s edge and 1.2 m above the floor by the Sony PCM-D100. The recording was carried out during heavy traffic hours during a workday. The traffic noise material was used in a previous study, which was carried out to study the effects of reverberation time and TNL on classroom English listening comprehension (Chen and Ou, 2021).

### Cognitive task

English reading comprehension task (ERCT) was evaluated in the experiment. The accuracy rate was calculated. The reading materials were selected from College English Test Band 4 (CET-4) examinations from 2008 to 2012. CET-4 was an authoritative examination to evaluate English proficiency for college students in China, ensuring consistency in reading difficulty between reading materials.

All ERCT materials were of similar lengths (542–675 words). After reading, a test sheet with five questions was used to assess reading comprehension performance. For each question, there were four choices, with only one correct answer. Reading materials and questions were paper-based. Participants were allowed to return to the text to find answers at any time according to their own needs, which was consistent with the actual reading comprehension work.

### Experimental procedure

Twenty-two undergraduate students (12 males and 10 females) from Huaqiao University (China) were recruited for this study. Based on the research on physiological parameters, the large effect size was 0.4 and the significance level was 0.05 (Thom, 2004; Qin et al., 2014). The sample size was calculated by G*Power Software. According to the results of G*Power, 10 samples were needed per group. Ten participants (five males and five females) were recruited for the group of absent plants and 12 participants (seven males and five females) for the group of present plants. All 22 participants performed the reading comprehension task under 5 acoustic conditions. Similar sample sizes were also used in previous studies (Hongyu et al., 2020; Min et al., 2021). Participants were native Chinese speakers and were tested individually. None of the participants reported any hearing or visual difficulties. Participants’ information was collected before the formal experiment, such as gender, noise sensitivity, English proficiency, and plant preference.

**Figure 3:**
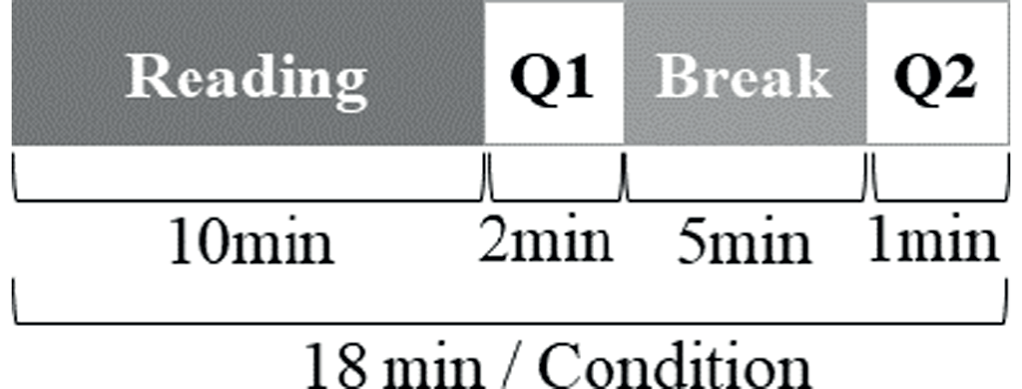
Experimental procedures for each condition.

As shown in Figure 3, the experimental procedure includes the following three stages:

Preparation stage: Individual preparative sessions took about 25 min, including the Tobii Pro Glasses 2 wearing and calibrating and the home office environment adapting. Instructions for experimental tasks were given before using the eye-tracking device.

Reading comprehension stage: Participants randomly conducted the given paper-based ERCT tasks under five acoustic conditions. In each condition, they were asked to complete the reading comprehension task within 10 min. A questionnaire Q1 was requested to be filled in at the end of each condition to obtain participants’ perception of mental workload and environmental quality during reading comprehension. Specific content and scoring methods were collected in Table 2.

**Table 2:** Descriptions of key aspects and sub-factors.

Key aspects	Sub-factors	Description
Work performance	Accuracy rate	Percentage of the correct answer in ERCT task. The scale is 0 to 100%
	Average pupil diameter (APD)	The average pupil diameter of all whole-fixation samples in the sound intervention stage, with the unit of millimeters. A larger pupil diameter indicates a higher cognitive load (Ahern and Beatty, 1979)
	Total amplitude of saccades (TAS)	The total amplitude of all saccades in the sound intervention stage, with the unit of degrees. Fewer saccade amplitudes indicate more focusing concentration (Yuji et al., 2009)
Mental workload	Mental workload	Mental workload was assessed by using the NASA Task Load Index (Hart and Staveland, 1988). In this study, five aspects (mental demand, temporal demand, effort, frustration, and perceived performance) of the NASA-TLX were used and each aspect was assessed by a 7-point scale (from 1 = very low to 7 = very high) in the questionnaire Q1. Specifically, mental demand represented the demands of the ERCTs on participants’ mental and perceptual activities, that was, the degree of task difficulty perceived by the participants. Temporal demand assessed whether participants rated time as slow or fast as they completed the task. The results of the Effort represented how much effort was required to achieve the highest task performance during the completion of the ERCT. Frustration measured the participant’s level of negative emotion when performing the ERCTs, such as insecure, discouraged, irritated, stressed, and annoyed. Perceived performance represented the degree of satisfaction with their performance in accomplishing the ERCT. The sum score of these five items was finally referred to as the results of participants’ mental workload, ranging from 7 to 35. The higher scores represented a higher mental workload
Feelings about the acoustic environment	Sound disturbance	Feelings about sound disturbance and acoustic satisfaction were surveyed with a 7-point Likert scale (Q1) after reading comprehension, from 1 (extremely low degree) to 7 (extremely high degree)
	Acoustic satisfaction	
Feelings about the non-acoustic environment	Layout satisfaction	Feelings about satisfaction regarding layout, thermal, lighting, and air quality were surveyed by Q1 with a 7-point Likert scale after reading comprehension, from 1 (extremely low degree) to 7 (extremely high degree)
	Thermal satisfaction	
	Lighting satisfaction	
	Air quality satisfaction	
Short-time break	Mental fatigue recovery (MFR)	Mental fatigue, visual fatigue, anxiety, and unfriendly were surveyed by Q1 and Q2 with a 7-point Likert scale, from 1 (extremely low degree) to 7 (extremely high degree). MFR, VFR, AR, and UR were calculated by the corresponding variable differences between Q1 and Q2. Higher scores represented higher levels of recovery
	Visual fatigue recovery (VFR)	
	Anxiety recovery (AR)	
	Unfriendly recovery (UR)	

A total of 5-min short-term break stage: After each condition, a 5-min break in a quiet environment was provided. Participants wearing the eye-tracking device could freely move about in the home office. At the start and end of each short-term break, they were asked to answer the questionnaire Q2 to obtain the perceived recovery effects. Specific content and scoring methods were collected in Table 2.

### Independent variables

Indoor environment quality is essential for participants’ work performance, mental workload, feelings about the acoustic environment, feelings about the non-acoustic environment, and short-term break recovery. Descriptions of the above five fundamental aspects and their corresponding sub-factors are listed in Table 2. The first four key aspects were assessed for the ERCT, including work performance, workload, feelings about the acoustic environment, and the non-acoustic environment, whereas accuracy rate, APD, and TAS were employed to show participants’ work performance.

**Figure 4:**
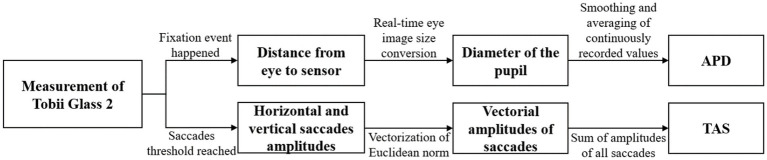
The process of data obtaining and calculation.

To evaluate eye movements, Tobii Pro Lab software was used to parse data into APD and TAS. All the data from the first gaze event to the final five choices were involved. The tracking quality for participants was effective and the tracking ratio across the acoustic conditions was higher than 80%. Tobii I-VT fixation filter processed and classified the gaze data into fixations and saccades. An angular velocity of 30 degrees per second was set as the limit, and eye movements less than that were classified as fixations, while those greater than that were classified as saccades. Only when the fixation event happened, the pupil diameter was measured and recorded. The eyeball model used by Tobii glass 2 can provide the distance data from the eye to the sensor. The built-in calculation model of the eye tracker can calculate the diameter of the pupil by calculating the pupil size on the real-time eye image and multiplying it by a conversion coefficient. Then the pupil diameter changes during the fixation event were smoothed using a 5-point moving average filter and recorded continuously (Van Der Meer et al., 2010), and the APD was obtained after averaging. Saccades thresholds were a minimum duration of 22 ms, and a peak velocity threshold of 40°/s. The sum of vectorial amplitudes of saccades generated by the total numbers of saccades was calculated as the total vector amplitude of saccades (TAS) (Nicola and Campagne, 2018; Jang et al., 2020), where the vectorial amplitude of a saccade was defined as the Euclidean norm of the horizontal and vertical amplitudes (Holland and Oleg, 2011). The process of data obtaining and calculation was summarized in Figure 4.

Sound disturbance and acoustic satisfaction, two sub-factors of the acoustic environment, were used to reveal participants’ feelings about the acoustic environment. Layout, thermal, lighting, and air quality satisfaction play vital roles in indoor environmental quality and usually show participants’ feelings about the non-acoustic environment. MFR, VFR, anxiety recovery (AR), and unfriendly recovery (UR) were sub-factors to show the restoration effects of short-term breaks. MFR, VFR, and AR measured feelings about the participants during the ERCT, and the UR referred to the feelings about the environment.

### Data analysis

Mixed analysis of variances (ANOVAs) was conducted to analyze the interaction effects of plants and TNLs on participants’ work performance (accuracy rate, APD, and TAS), with work performance as a dependent variable while TNL as a within-subjects independent variable and plants as a between-subjects factor independent variable. Mixed ANOVAs revealed the main effect of plants of TNLs on work performance. For each TNL, Independent-sample *t*-tests were employed to determine differences between conditions without and with plants in work performance. Repeated measures of analysis of variance (RMANOVA) tests were employed to determine differences in work performance, with TNL as the independent variable. RMANOVAs were tested for conditions with plants and conditions without plants separately. The significant level is expressed as *p*-value. The *p*-value<0.05 means a statistically significant difference. The *p*-value<0.10 means a marginally significant difference.

To determine the interaction effect between perceived evaluations at different TNLs and plants, generalized estimating equation (GEE) tests were performed with repeated measures using ordinal logistic function and an exchangeable correlation structure. GEE was performed with TNL as within-subjects independent variables and plants as between-subjects factor independent variables. Every condition was used as the baseline to assess whether the association between TNLs and perceived evaluations differ by plants. Analyses were conducted separately for 10 different conditions. The significant level of interaction effects was expressed as *p*-values. It was accepted if four orthogonal conditions (2 TNLs * 2 plants) had significant effects on each other (*p*-value<0.1). Three types of interaction effects were considered: (1) different trend with the increase of TNL, (2) similar trend with the increase of TNLs but further growth or decrement between conditions with and without plants, and (3) differences between conditions with and without plants at the same TNL. Odds ratios (ORs) were also included for interaction to compare the risk level. When an OR was greater than 1, an increased TNL risk was reported (i.e., OR = 1.50 indicated a 50% increased risk), and vice versa. For perceived evaluation parameters, participants’ workload, feelings about the environment, and short-term break recovery (see Table 2) were considered here. Mann–Whitney *U*-tests were also employed to determine differences in items between conditions without and with plants for each TNL. Friedman tests with TNL as the independent variable were employed to determine if there were differences in items among different TNLs under conditions without and with plants. All data were analyzed using IBM SPSS Statistics Software.

## Effects of visual and acoustic conditions on reading comprehension

### Effects of plants and TNLs on work performance

**Figure 5:**
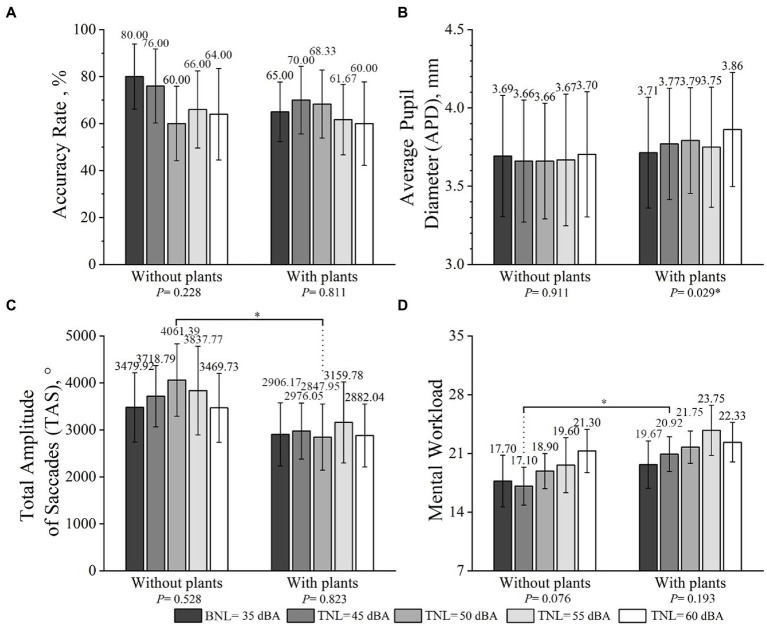
Mean accuracy rate **(A)**, APD **(B)**, TAS **(C)**, and workload **(D)** at different TNLs (error bars define 95% CI) in the presence and absence of plants. * represents a significant effect of plants in conditions at the same TNL.

The results of participants’ mean accuracy rate, APD, and TAS of ERCT at different TNLs in the presence and absence of plants are shown in Figure 5. A consistent trend is observed in the participants working in home offices with plants which have lower accuracy rates, higher APD, and lower TAS than those working in home offices without plants.

#### Interaction effects between plants and TNLs

In terms of interaction between plants and TNLs on work performance, mixed ANOVA tests revealed no significant interaction between plants and TNLs on accuracy rate [*F*(4,80) =0.730, *p*-value >0.100, $\eta_{P}^{2}$ =0.035], APD [*F*(4,80) =1.064, *p*-value >0.100, $\eta_{P}^{2}$ =0.051], and TAS [*F*(4,80) =0.613, *p*-value >0.100, $\eta_{P}^{2}$ =0.030] (see Figure 5). In addition, there was no significant effect for TNLs on accuracy rate [*F*(4,80) =1.151, *p*-value >0.100, $\eta_{P}^{2}$ =0.054], APD [*F*(4,80) =1.621, *p*-value >0.100, $\eta_{P}^{2}$ =0.075], and TAS [*F*(4,80) =0.767, *p*-value >0.100, $\eta_{P}^{2}$ =0.037]. Similarly, no significant effect was observed for plants on accuracy rate [*F*(1,20) =0.473, *p*-value >0.100, $\eta_{P}^{2}$ =0.023] and APD [*F*(1,20) =0.160, *p*-value >0.100, $\eta_{P}^{2}$ =0.008]. It is worth noting that the TAS of participants working in the absence of plants was higher than that in the presence of plants at a marginal significance level [*F*(1,20) =3.572, *p*-value =0.073, $\eta_{P}^{2}$ =0.152] (see Figure 5).

#### Effects of plants

In terms of the effects of plants on participants’ work performance, independent-sample *t*-tests revealed no significant difference in accuracy rates and APD (*p*-value >0.100) between conditions without and with plants. However, the independent-sample *t*-tests results demonstrated: (1) the TAS for participants in “NP_T_50” was significantly greater than in “P_T_50” (*p*-value <0.050); (2) participants’ TAS in “NP_T_45” was higher than that in “P_T_45” at a marginal significance level (*p*-value = 0.094).

#### Effects of TNLs

In terms of the effects of TNLs on participants’ work performance, RMANOVA tests revealed that there was no significant difference among five TNLs in accuracy rates [*F*(4,36) =1.481, *p*-value>0.100, $\eta_{P}^{2}$ =0.141], APD [*F*(4,36) =0.244, *p*-value>0.100, $\eta_{P}^{2}$ =0.026], and TAS [*F*(4,36) = 0.809, *p*-value>0.100, $\eta_{P}^{2}$ =0.082] when the plants were absent. Similarly, no significant difference was found among five TNLs in accuracy rates [*F*(4,44) =0.396, *p*-value>0.100, $\eta_{P}^{2}$ =0.035] and TAS [*F*(4,44) =0.379, *p*-value>0.100, $\eta_{P}^{2}$ =0.033] when the plants were present. A significant difference, however, was demonstrated in APD among five TNLs when the plants were present [*F*(4,44) =2.991, *p*-value <0.05, $\eta_{P}^{2}$ =0.214]. An increasing trend in APD was also observed with the increasing TNLs when the plants were present (see Figure 5).

### Effects of plants and TNLs on mental workload

The results of participants’ mental workload of ERCT at different TNLs both when the plants were present and absent are shown in Figure 5D. A consistent trend was observed in that participants’ mental workloads increasing with TNLs, and participants working in home offices with plants took on more mental workload than without plants.

#### Interaction effects between plants and TNLs

Next, the interaction between plants and TNLs on participants’ mental workload were examined. The GEE results revealed a significant association between plants and TNLs on mental workload (Wald χ2 = 24.961, *p*-value <0.010) (see Figure 5D). More specifically, there was a crossover interaction between plants and TNLs (35 and 45 dBA) to the effect that in the absence of plants, the higher TNL = 45 dBA decreased mental workload, but in the presence of plants, the lower TNL = 35 dBA decreased mental workload (*p*-value<0.050). Furthermore, participants at TNL = 45 dBA were associated with a significantly higher risk of reporting high layout satisfactions when the plants were present than absent (OR = 5.151, *p*-value<0.050).

#### Effects of plants

To evaluate plants’ effects on participants’ mental workload, Mann–Whitney *U*-tests were carried out. A significant difference was found between the presence and absence of plants when the TNL was at 45 dBA (*p*-value <0.050) (shown in Figure 5D). Additionally, marginally significant differences in mental workloads were found between the presence and absence of plants when TNLs were at 50 dBA (*p*-value = 0.063) and 55 dBA (*p*-value = 0.069) (see Figure 5D).

#### Effects of TNLs

Next, the effects of TNLs on participants’ mental workload were assessed. No significant difference was found among all acoustic conditions in either the absence or presence of indoor plants based on Friedman tests (*p*-value >0.050) (see Figure 5D).

### Effects of plants and TNLs on feelings about the acoustic environment

**Figure 6:**
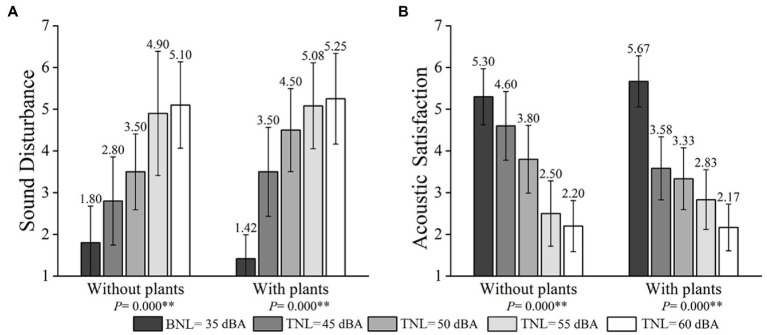
Mean sound disturbance **(A)** and acoustic satisfaction **(B)** at different TNLs (error bars define 95% CI) in the presence and absence of plants.

In the presence and absence of plants, participants’ sound disturbance and acoustic satisfaction of ERCT at different TNLs are shown in Figure 6. A consistent trend could be observed in all conditions that participants’ sound disturbance increased and acoustic satisfaction decreased with the increase of TNLs. Table 3 summarizes the significant interaction effects of plants and TNLs on participants’ feelings about the acoustic environment.

**Table 3:** GEE results of interaction effects on short-time breaks between plants and TNLs.

Reference condition		Interactive condition		AR		UR	
TNL (dBA)	Plants	TNL, dBA	Plants	ORs	*P*-value	ORs	*P*-value
35	No	55	Yes	11.270	0.003**	17.059	0.001**
			No	7.660	0.015*	16.599	0.002**
		60	Yes	20.647	0.000**	44.506	0.000**
			No	13.867	0.002**	22.586	0.001**
35	Yes	55	Yes	17.859	0.000**	22.981	0.000**
			No	12.139	0.002**	22.362	0.000**
		60	Yes	32.719	0.000**	59.957	0.000**
			No	21.975	0.000**	30.427	0.000**
45	No	60	Yes	19.991	0.000**		
			No	13.426	0.002**		
45	Yes	60	Yes	9.407	0.003**		
			No	6.318	0.019*		

Only the results with significant effect (*P*-value < 0.05) are given. *P-value < 0.050; **P-value < 0.010. ORs: if the OR value is greater than 1, the larger OR value indicates a more negative effect of traffic noise on participants; if the OR value is less than 1, the smaller OR value indicates a more negative effect of traffic noise on participants.

#### Interaction effects between plants and TNLs

The interaction between plants and TNLs on participants’ feelings about the acoustic environment (sound disturbance and acoustic satisfaction) were evaluated. The GEE’s results revealed a significant interaction between plants and TNLs on sound disturbance (Wald χ2 = 54.606, *p*-value <0.010) (see Figure 6A) and acoustic satisfaction (Wald χ2 = 60.664, *p*-value <0.010) (see Figure 6B).

For sound disturbance, as shown in Table 4, (1) when the plants were present at 35 and 45 dBA, the increase of sound disturbance was significantly associated with TNL (OR = 32.099, *p*-value <0.001). Significant associations were also observed between 35 and 50 dBA (OR = 89.106, *p*-value <0.001), 35 and 55 dBA (OR = 189.885, *p*-value <0.001), 35 and 60 dBA (OR = 272.958, *p*-value <0.001). In comparison, the increased risk of sound disturbance was lower if the plants were absent at 45 dBA (OR = 15.034), 50 dBA (OR = 32.907), and 60 dBA (OR = 180.225) (*p*-value <0.010). However, this trend was reversed between 35 dBA and 55 dBA (OR = 200.798, *p*-value <0.001). (2) When the plants were present at 45 and 55 dBA, the increase of sound disturbance was significantly associated with TNL (OR = 5.916, *p*-value <0.050). A significant association was also observed between 45 and 60 dBA (OR = 8.504, *p*-value <0.050). In comparison, the increased risk of sound disturbance was lower if plants were absent at 60 dBA (OR = 5.615, *p*-value <0.050).

**Table 4:** GEE results of interaction effects on feelings about the acoustic environment between plants and TNLs.

Reference condition		Interactive condition		Sound disturbance		Acoustic satisfaction	
TNL (dBA)	Plants	TNL (dBA)	Plants	ORs	*P*-value	ORs	*P*-value
35	*No*	45	Yes	11.581	0.004**	0.059	0.001**
			No	5.424	0.050*	0.247	0.093
		50	Yes	32.149	0.000**	0.037	0.000**
			No	11.873	0.005**	0.088	0.005**
		55	Yes	68.510	0.000**	0.019	0.000**
			No	72.448	0.000**	0.009	0.000**
		60	Yes	98.483	0.000**	0.005	0.000**
			No	65.025	0.000**	0.007	0.000**
35	Yes	45	Yes	32.099	0.000**	0.037	0.000**
			No	15.034	0.003**	0.155	0.023*
		50	Yes	89.106	0.000**	0.023	0.000**
			No	32.907	0.000**	0.055	0.001**
		55	Yes	189.885	0.000**	0.012	0.000**
			No	200.798	0.000**	0.006	0.000**
		60	Yes	272.958	0.000**	0.003	0.000**
			No	180.225	0.000**	0.004	0.000**
45	No	55	Yes	12.630	0.002**		
			No	13.356	0.002**		
		60	Yes	18.156	0.000**	0.021	0.000**
			No	11.988	0.003**	0.028	0.000**
45	Yes	55	Yes	5.916	0.018*		
			No	6.256	0.020*		
		60	Yes	8.504	0.018*	0.087	0.002**
			No	5.615	0.028*	0.117	0.008**
50	No	60	Yes			0.059	0.001**
			No			0.079	0.003**
50	Yes	60	Yes			0.139	0.011*
			No			0.187	0.036*

Only the results with significant effect (*P*-value < 0.05) are given. *P-value < 0.050; **P-value < 0.010. ORs: if the OR value is greater than 1, the larger OR value indicates a more negative effect of traffic noise on participants; if the OR value is less than 1, the smaller OR value indicates a more negative effect of traffic noise on participants.

For acoustic satisfaction, as shown in Table 4, (1) when the plants were present at 35 and 45 dBA, the decrease in acoustic satisfaction was significantly associated with TNL (OR = 0.037, *p*-value <0.001). Significant associations were also observed between 35 and 50 dBA (OR = 0.023, *p*-value <0.001), 35 and 55 dBA (OR = 0.012, *p*-value <0.001), 35 and 60 dBA (OR = 0.003, *p*-value <0.001). In comparison, the decreased risk of acoustic satisfaction was lower if the plants were absent at 45 dBA (OR = 0.155), and 50 dBA (OR = 0.055) (*p*-value <0.050). However, this trend was reversed between 35 and 55 dBA (OR = 0.006, *p*-value <0.001). (2) When the plants were present at 45 and 60 dBA, the decrease in acoustic satisfaction was significantly associated with TNL (OR = 0.087, *p*-value <0.010). (3) When the plants were present at 50 and 60 dBA, the decrease in acoustic satisfaction was significantly associated with TNL (OR = 0.139, *p*-value <0.050).

#### Effects of plants

To assess plants’ effects on participants’ sound disturbance, Mann–Whitney *U*-tests were performed. No significant difference was found between the presence and absence of plants (see Figure 6A). Additionally, the acoustic satisfaction at 45 dBA TNL was marginally significantly higher in the absence of plants than that in the presence of plants (*p*-value = 0.083) (see Figure 6B).

#### Effects of TNLs

Furthermore, the effects of TNLs on participants’ feelings about the acoustic environment were examined. The results showed significant differences among all acoustic conditions both when the plants were present and absent (*p*-value <0.010) (see Figure 6). It indicates that the influence of TNLs on participants’ feelings about the acoustic environment is more potent compared with the impact of plants.

### Effects of plants and TNLs on feelings about the non-acoustic environment

#### Interaction effects between plants and TNLs

**Figure 7:**
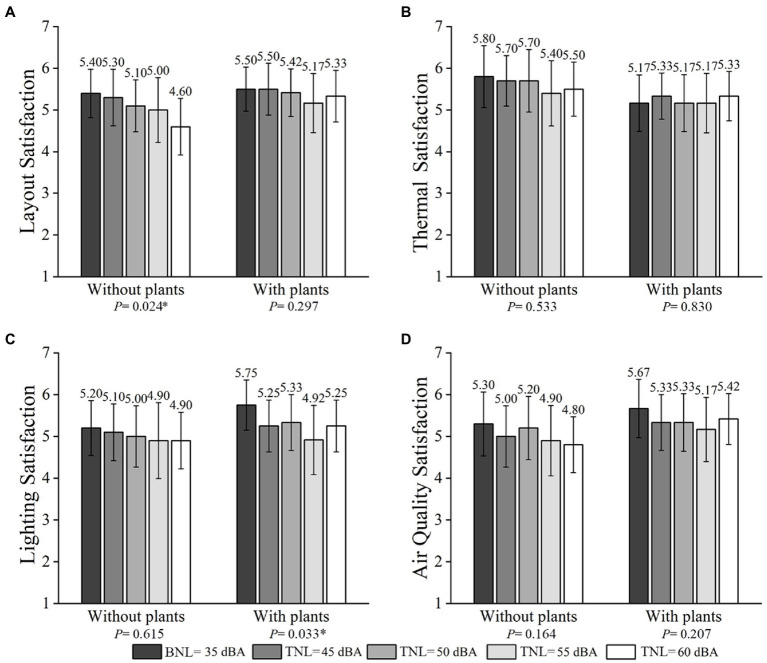
Mean layout **(A)**, thermal **(B)**, lighting **(C)**, and air quality environmental satisfaction **(D)** at different TNLs (error bars define 95% CI) in the presence and absence of plants.

The results of participants’ layout, thermal, lighting, and air quality satisfaction of ERCT at different TNLs, both when the plants were present and absent are presented in Figure 7 to reveal the interaction between plants and TNLs on participants’ feelings about the non-acoustic environment. The GEE results revealed a significant association between plants and TNLs on layout satisfaction (Wald χ^2^ = 33.296, *p*-value <0.010), whereas no significant interaction was found between plants and TNLs on thermal, lighting, and air quality satisfaction (*p*-value >0.100). More specifically, participants at TNL = 60 dBA had a significantly higher risk of reporting high layout satisfaction when the plants were present than when the plants were absent (OR = 4.060, *p*-value<0.050).

#### Effects of plants

In terms of plants’ effects on participants’ feelings about the non-acoustic environment, no significant difference was found between the presence and absence of plants based on Mann–Whitney *U*-tests results (*p*-value>0.100) (see Figure 7).

#### Effects of TNLs

Regarding the effects of TNLs on participants’ feelings about the non-acoustic environment, no significant difference in thermal, lighting, and air quality satisfaction was observed when the plants were absent based on Friedman tests. No significant difference in layout, thermal, and air quality satisfaction was found when the plants were present (*p*-value >0.100) (see Figure 7). It is worth noting that a significant difference in layout satisfaction was demonstrated among TNLs when the plants were absent (*p*-value <0.05) (see Figure 7A), and a significant difference in lighting satisfaction was found among TNLs when the plants were present (*p*-value <0.05) (see Figure 7C). These results showed that participants’ layout satisfactions decreased with the increasing TNLs when the plants were absent. Participants’ lighting satisfaction was highest at 35 dBA and lowest at 55 dBA when the plants were present.

## Effects of visual and acoustic conditions on short-time breaks

**Figure 8:**
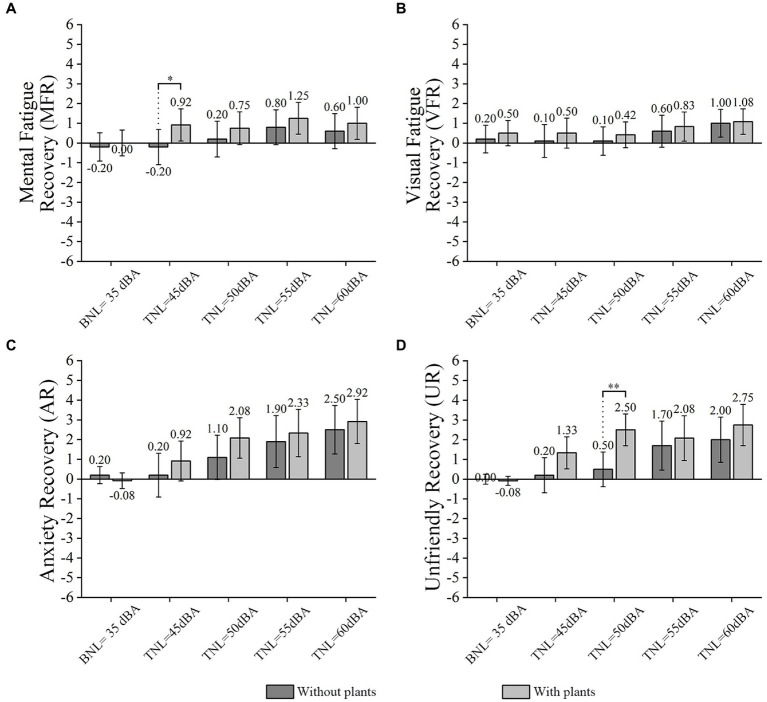
Mean MFR **(A)**, VFR **(B)**, AR **(C)**, and UR **(D)** at different TNLs (error bars define 95% CI) in the presence and absence of plants. * represents a significant effect of plants in conditions at the same TNL.

The results of participants’ MFR, VFR, anxiety recovery (AR), and unfriendly recovery (UR) in short-term breaks are presented in Figure 8. The horizontal axis in Figure 8 represents the tested acoustic condition before a short-term break. A quiet environment with plants was tended to promote MFR, VFR, AR, and UR compared with that without plants. Table 3 summarizes the significant interaction effects of plants and TNLs on participants’ short-term breaks.

### Interaction effects between plants and TNLs

In terms of the interaction between plants and TNLs on short-term breaks, the GEE’s results revealed significant interactions between plants and TNLs on AR (Wald χ^2^ = 33.984, *p*-value <0.010, see Figure 8C) and UR (Wald χ^2^ = 40.409, *p*-value <0.010, see Figure 8D), and a marginally significant interaction between plants and TNLs on MFR (Wald χ^2^ = 15.391, *p*-value =0.081, see Figure 8A). In addition, there was no significant interaction between plants and TNLs on VFR (Wald χ^2^ = 10.976, *p*-value>0.100, see Figure 8B).

For AR, as shown in Table 3, (1) when the plants were present at 35 dBA and 55 dBA, the increase of AR was significantly associated with TNL (OR = 17.859, *p*-value <0.001). A significant association was also observed between 35 and 60 dBA (OR = 32.719, *p*-value <0.001). In comparison, the increased risk of AR was lower if plants were absent at 55 dBA (OR = 12.139) and 60 dBA (OR = 21.975) (*p*-value <0.010). (2) when the plants were present at 45 and 60 dBA, the increase of AR was significantly associated with TNL (OR = 9.407, *p*-value <0.010). In comparison, the increased risk of AR was lower if plants were absent at 60 dBA (OR = 6.318, *p*-value <0.050).

For UR, as shown in Table 3, (1) when the plants were present at 35 and 55 dBA, the increase of UR was significantly associated with TNL (OR = 22.981, *p*-value <0.001). A significant association was also observed between 35 and 60 dBA (OR = 59.957, *p*-value <0.001). In comparison, the increased risk of UR was lower if plants were absent at 60 dBA (OR = 30.427) (*p*-value <0.010).

### Effects of plants

In terms of the effects of plants on participants’ short-term breaks, Mann–Whitney *U*-tests revealed no significant difference in VFR and AR between conditions without and with plants (*p*-value >0.100). However, the Mann–Whitney *U*-tests results showed that the MFR for participants in “P_T_45” was significantly higher than that in “NP_T_45” (*p*-value <0.050, see Figure 8A), and participants’ UR in “P_T_45” was higher than that in “NP_T_45” at a marginal significance level (*p*-value =0.076, see Figure 8D). Furthermore, the UR for participants in “P_T_50” was significantly higher than that in “NP_T_50” (*p*-value <0.010, see Figure 8D).

## Discussion

The above analysis demonstrates an interaction between indoor plants and TNLs in English reading comprehension and short-term breaks in home offices. The results provide helpful guidance for the quality improvement of indoor environments, especially for the two-sided performance of plants. It should be kept in mind that for a positive project of work performance, the simultaneous analysis of visual and acoustic conditions may be needed. Although only ERCTs has been tested and analyzed in our study, traffic noise and indoor plants’ adverse effects on improved work performance are well demonstrated.

It is also interesting that TNLs can be divided into three grades of small (35 dBA), medium (45 and 50 dBA), and large (55 and 60 dBA), according to the experimental results. For example, 35 dBA BNL is always the superior condition for ERCT, since participants’ work performance and perceived indoor environment qualities under 35 dBA BNL are better than the other four tested TNL conditions. Significant differences in ERCT and short-term breaks are mostly found at 45 and 50 dBA TNL between the absence and presence of plants, as shown in Figures 5–8. Participants’ work performance and perceived indoor environment qualities decrease with increasing TNLs. The significant interactions have been identified between small, medium and large TNL, as shown in Tables 3, 4.

In terms of small TNL, it is in line with some previous investigations (Kalevi et al., 2017; Van den Bogerd et al., 2021) but inconsistent with the results of Ruth et al. (2011), who have argued that the presence of plants is more likely to improve the reading span task performance than the absence of plants because of the mediated arousal level introduced by indoor plants. Further looking into Ruth’s study (Ruth et al., 2011), it can be seen that the cognitive task is the main reason why the presence of plants improves work performance more than the absence of plants. The relationship between task performance and arousal is inverted-U, and the mediate arousal provides optimal performance (Matthew et al., 2015). The mediated arousal level increases with the task’s difficulty (Yerkes and Dodson, 1908), which indicates that the ERCT needs more cognitive abilities and requires a higher arousal level to achieve optimal performance than the reading span task. Based on previous studies, viewing plants could activate parasympathetic nervous system activity (Gladwell et al., 2012; Lee et al., 2015), related to low arousal (Robert, 2004), indicating that the arousal level is lower when the plants are present than the plants are absent. The arousal level provided by plants is closer to the best required by the reading span task (a simple task). The arousal level without plants is more relative to the optimal arousal level required for ERCT (a difficult task). These findings have significant implications for understanding why plants increase work performance in some studies but not in others.

In terms of medium and large TNLs, higher sound disturbance and lower work performance are found in conditions with plants than those without plants, especially at medium TNLs, as shown in Figures 5, 6. It should also be noted that APDs are significantly affected by TNLs only when indoor plants are present, as shown in Figure 5B. Compensatory efforts are motivated to narrow the deviation between the current and the expected (mediated) arousal levels (Sleegers et al., 2021). As expected, higher mental workloads with plants are also reported in this study (see Figure 5D. Additional compensatory efforts on the ERCT are applied in the presence of plants. Other attentional resources generated by compensatory measures in the brain are also used to process the ERCT. In this way, fewer attentional resources can be used to suppress the brain’s automatic processing of traffic noise when the plants are present. It is expected that participants will perceive more sound disturbance and further impair the ERCT performance. This study is the first comprehensive investigation of the interactive relationship between indoor plants and TNLs on ERCT performance in home offices. The findings are worth considering in future studies.

An important finding is that ERCT performance decrement depends on visual and acoustic conditions. To better analyze the relationships between TNLs and ERCT performance, the work performance and mental workload results in Figure 5 will be considered. Some interesting points can be observed. As shown in Figure 5, there is no denying that ERCT performances decrease with the increase of TNLs in all conditions. However, when indoor plants are present at 55 dBA TNL, participants’ mental workload has already reached the top point (see Figure 5D). The results must be interpreted cautiously in the presence of plants because a relatively decreased APD and a relatively increased TAS at 55 dBA TNL have captured our attention. Based on the results and analysis of this study, the reason can be ascribed to the following aspects: (1) cognitive overload: cognitive overload leads to the pupil diameters decrement (Granholm et al., 1996); (2) blink rate decrement: the increase of mental workload is associated with the decrease of blink rates which is associated with visual fatigue (Morris and Gerald, 1972; Ledger, 2013), and a high TAS is triggered to counteract the reduction in blink rates (Cardona and Quevedo, 2013). Although the study did not show the results of blink rates, it did substantiate the effect of traffic noise on eye movement, and blink rates could be considered in future studies.

In addition, as reported by most previous studies (Ulrich, 1983; Ulrich et al., 1991; Sin-Ae et al., 2017), short-term breaks with plants in this study also provide more psychological recovery than without plants. For example, participants having a short break in home offices with plants perform a significantly higher MFR at 45 dBA TNL (see Figure 8A) and a significantly higher UR at 50 dBA TNL (see Figure 8D) than those resting without plants. To sum up, despite the potential negative effects indoor plants may have on work performance, they positively affect short-term break recovery.

The limitation of this study is that only one type of plant was used in this study, and the sample size was still relatively small as a whole. In order to more accurately quantify the effect relationship, the sample size, plant species, and plant quantities are needed to expand in the follow-up study.

## Conclusion

This study has explored the interaction effects of indoor plants and TNLs on ERCTs and short-term breaks in home offices. The first goal was to observe participants’ work performance and environmental perceptions of the ERCT, including accuracy rates, eye movements, mental workload, and feelings about the environment. The second aim was to further analyze the effects of ERCT on a 5-min short-term break by comparing the MFR, VFR, anxiety recovery, and unfriendly recovery. Two visual conditions (with and without plants) and five acoustic conditions (BNL = 35 dBA, TNL = 45, 50, 55, and 60 dBA) were included in this study. In terms of both the reading comprehension stage and short-term breaks, when the sound pressure level of traffic noise does not exceed 50 dBA, placing indoor plants in home offices is beneficial for the work based on English reading comprehension. When the traffic noise exceeds 50 dBA, indoor plants have ignorable effects on participants. More detailed findings are listed as follows:

1. The comparisons between indoor plants’ absence and presence reveal that at 45 dBA TNL, participants working with plants have significantly higher mental workloads than those working in home offices without plants. At 50 dBA TNL, participants working with plants have a significantly lower TAS than those working in home offices without plants. After reading at 45 dBA TNL, participants breaking with plants have significantly higher mental fatigue recoveries than those breaking without plants. After reading at 50 dBA TNL, participants breaking with plants have significantly higher unfriendly recoveries than those breaking without plants.
2. The effects of TNLs on participants differ in the presence of plants. When the indoor plants are present, significantly increased participants’ APDs and decreased lighting satisfaction is found with the increase of TNLs. No significant effect of TNLs on participants’ APDs and lighting satisfaction is found when the indoor plants are absent.
3. Plants interact with TNLs (35 and 45 dBA) to the effect that the higher TNL = 45 dBA increases mental workload with plants’ presence, but with the absence of plants, the higher TNL = 45 dBA decreases mental workload.
4. Participants’ layout satisfaction decreases with the increase of TNLs in conditions without plants, while no significant effect is found in conditions with plants. There is a substantial interaction between indoor plants and TNLs to the effect that the presence of plants is more likely to be reported a higher layout satisfaction at 60 dBA TNL than the absence of plants.
5. The interaction effects of indoor plants and traffic noise on ERCT differ by the range of TNLs. When TNLs increase from 35 to 60 dBA, participants’ feelings about the acoustic environment worsen drastically with TNLs in the absence of plants, while they get worse with a slow rate in the presence of plants, as well as when TNLs increase from 35 to 50 dBA. When TNLs increase from 35 to 55 dBA, they worsen drastically with TNLs in plants’ presence, as well as when TNLs increase from 45 dBA to a higher level.

## Data availability statement

The raw data supporting the conclusions of this article will be made available by the authors, without undue reservation.

## Ethics statement

Ethical review and approval was not required for the study on human participants in accordance with the local legislation and institutional requirements. The patients/participants provided their written informed consent to participate in this study.

## Author contributions

YZ: conceptualization, methodology, software, validation, formal analysis, investigation, data curation, writing – original draft, and visualization. DO: conceptualization, methodology, investigation, resources, data curation, writing – review and editing, visualization, supervision, project administration, and funding acquisition. QC: investigation, data curation, and validation. SK: conceptualization, methodology, software, validation, and writing – review and editing. GQ: visualization and validation. All authors contributed to the article and approved the submitted version.

## Funding

This work was supported by the Natural Science Foundation of Fujian Province of China (2018J01070), State Key Lab of Subtropical Building Science, South China University of Technology (2022ZA02), Social Science Planning Project of Fujian Province of China (FJ2021B075), and National Natural Science Foundation of China (51578252).

## Conflict of interest

The authors declare that the research was conducted in the absence of any commercial or financial relationships that could be construed as a potential conflict of interest.

## Publisher’s note

All claims expressed in this article are solely those of the authors and do not necessarily represent those of their affiliated organizations, or those of the publisher, the editors and the reviewers. Any product that may be evaluated in this article, or claim that may be made by its manufacturer, is not guaranteed or endorsed by the publisher.
